# Sheep Displayed No Clinical and Parasitological Signs upon Experimental Infection with *Babesia aktasi*

**DOI:** 10.3390/vetsci11080359

**Published:** 2024-08-08

**Authors:** Mehmet Can Ulucesme, Sezayi Ozubek, Munir Aktas

**Affiliations:** Department of Parasitology, Faculty of Veterinary Medicine, University of Fırat, Elazığ 23200, Türkiye; mculucesme@firat.edu.tr (M.C.U.); sozubek@firat.edu.tr (S.O.)

**Keywords:** *Babesia aktasi*, experimental study, sheep, pathogenicity

## Abstract

**Simple Summary:**

In this study, an experimental investigation was conducted to assess the pathogenicity of *Babesia aktasi* in immune-suppressed sheep. For this purpose, five immune-suppressed lambs under one year of age were infected by intravenous injection of fresh blood containing approximately 9.2% and 12% parasitemia of *B. aktasi*. Following parasite injection, the lambs were monitored daily for clinical and microscopic findings of babesiosis for 30 days. Throughout this period, no clinical and microscopic signs of babesiosis were observed in the lambs. Of the five recipient lambs, two tested negative for *B. aktasi* in nested PCR up to 30 days post-infection. Among the remaining three lambs, two were PCR positive on the first day, and the other one remained positive until the fourth day post-infection. DNA sequencing confirmed that the PCR positivity in the recipient lambs originated from the inoculum. These findings indicate that immune-suppressed lambs do not appear to be susceptible to infection with *B. aktasi*, which is highly pathogenic to immune-suppressed indigenous goats.

**Abstract:**

Our survey in the Mediterranean region of Türkiye revealed high prevalence of *Babesia aktasi* in goats, while no molecular evidence of the parasite was found in sheep grazing in the same pasture. We hypothesized that the parasite may not be infectious to sheep. To test this hypothesis, the present study was designed to evaluate the susceptibility of Akkaraman sheep breed to *B. aktasi* infection. Fifteen mL of fresh blood infected with *B. aktasi* was injected into immune-suppressed lambs (*n* = 5). The recipient lambs were monitored daily for clinical signs of babesiosis over 30 days, and blood was collected for microscopic and molecular diagnostic evaluation. The lambs did not display clinical and parasitological signs of babesiosis. Two out of five recipient lambs were nested PCR-negative for *B. aktasi* over 30 days post infection. Out of the remaining three lambs, two were PCR positive on the first day, and one recipient was positive until the fourth day post infection. DNA sequencing confirmed that the PCR positivity in the recipient lambs originated from the inoculum. These findings revealed that immune-suppressed sheep do not appear to be susceptible to infection with *B. aktasi* that is lethal to immune-suppressed indigenous goats.

## 1. Introduction

Parasitic diseases in the livestock industry have a major economic impact because of losses related to animal deaths and preventive control efforts [1]. Among these, babesiosis is a tick-borne hemoparasitic disease caused by intra-erythrocytic protozoa belonging to the phylum Apicomplexa and order Piroplasmida [2,3]. Over 100 species of *Babesia* that infect humans, domestic and wild mammals, and birds have been identified globally, with this number likely to increase as research expands to other vertebrate hosts [4]. The disease is characterized by high fever, anemia, jaundice, hemoglobinuria, and in severe cases, results in death, particularly in domestic ruminants [5,6,7]. Ovine babesiosis is a major threat to the health of small ruminants [5,6]. *Babesia ovis*, *B. motasi*, and *B. crassa* notably cause babesiosis in small ruminants, with *B. ovis* being the main pathogen of clinical disease in sheep [3,8,9,10,11]. Advances in molecular parasitology over the past two decades have heightened interest in blood protozoa from the piroplasmid lineage, leading to the discovery of novel *Babesia* species or genotypes in small ruminants, including *Babesia* Xinjiang, *Babesia lengau*-like, and *B. motasi*-like variants [3,10,12,13]. Recently, a novel *Babesia* sp. infecting indigenous goats was identified in the Mediterranean region of Türkiye [14]. It was subsequently isolated from a naturally infected goat and named *Babesia aktasi* [14]. An experimental study later demonstrated that *B. aktasi* induced typical clinical signs of babesiosis, including fever, anemia, icterus, and hemoglobinuria, ultimately leading to death in immune-suppressed indigenous goats [15].

Previous epidemiological studies on babesiosis in small ruminants revealed that *B. ovis*, *B. crassa*, *B. motasi*, *Babesia* Xinjiang, and *B. motasi*-like variants [*Babesia* sp. BQ1 (Lintan), *Babesia* sp. BQ1 (Ningxian)] were infective for both sheep and goats [10,12,16,17,18]. There is no information available on whether the newly identified *B. aktasi* in goats is infectious for sheep. In fact, our molecular survey which aimed to determine the frequency of *B. aktasi* in goats and sheep in the Mediterranean region of Türkiye indicated a high prevalence in local goats. However, the parasite was not detected in the sampled sheep grazing in the same flock with the goats in the region [19]. These epidemiological data strongly support the fact that *B. aktasi* may not be infective for sheep. But, these findings do not definitively indicate that the parasite does not establish an infection in sheep. It would be important to determine whether the presence of *B. aktasi*-infected sheep constitutes a risk factor for goat infection, as this could have significant implications for the epidemiology of caprine babesiosis caused by *B. aktasi*. Therefore, the present experimental study was designed to evaluate the susceptibility of the Akkaraman sheep breed to *B. aktasi* infection. Our findings were discussed in the context of epidemiology, transmission, and reservoir capacity for the causative agent of caprine babesiosis, *B. aktasi*.

## 2. Materials and Methods

### 2.1. Parasite (Babesia aktasi Stabilate)

*Babesia aktasi* stabilate used in this study was isolated from a naturally infected indigenous goat as previously described [14]. Specifically, during our field survey in Antalya Province, Türkiye, one goat was identified as infected with *B. aktasi* through molecular diagnostic tests. This goat was purchased and transported to our facility for the experimental study. The goat was splenectomized to increase the amount of parasites in circulation. When parasitemia reached approximately 1.9%, 20 mL of infected blood was passaged in another immune-suppressed goat. On the 13th day post-splenectomy, when parasitemia peaked, jugular venous blood was collected from the second goat into EDTA-coated vacutainer tubes. Packed infected red blood cells were cryopreserved with 8% dimethyl sulfoxide, and kept in liquid nitrogen. All experiment procedures were approved by the Fırat University, Animal Experiments Local Ethics Committee (session number: 2021/12).

### 2.2. Selection of Experimental Animals

**Figure 1:**
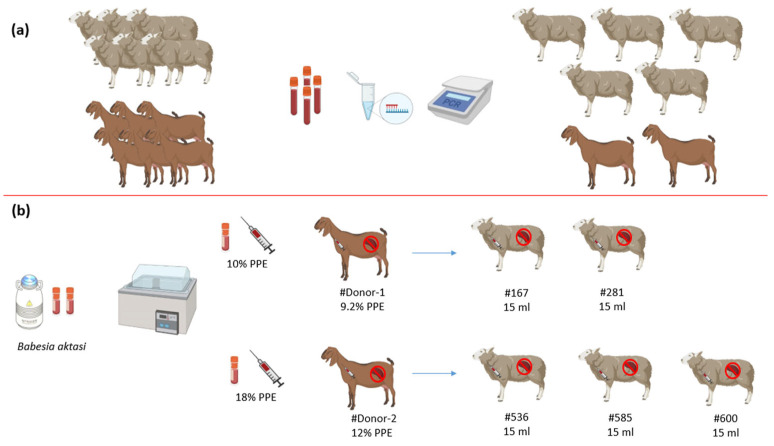
Representative schema of experimental infections. (**a**) Selection of the donors and lambs. (**b**) Infection of #Donor-1 and #Donor-2 with *B. aktasi* stabilates at 10% and 18% PPE (percentage of parasitized erythrocytes), respectively, and subsequent infection of the lambs with fresh blood infected with *B. aktasi* taken from #Donor-1 and #Donor-2.

Akkaraman breed lambs and indigenous goats, free from *Babesia, Anaplasma, and Theileria,* were used in this study. For this purpose, whole blood was collected in serum and EDTA tubes from the apparently healthy lambs and goats aged 4 to 7 months in a breeding farm located in a village of Elazığ Province, Türkiye. Peripheral thin blood smears from the ear vein of the same animals were prepared for the microscopic examination. Thin blood smears and DNAs isolated from EDTA blood samples were screened by microscopy and nested PCR for the presence of *Babesia* spp., *Theileria* spp., and *Anaplasma* spp., respectively [19,20]. For the amplification of *Anaplasma* spp., the primers Ec9/Ec12A [21] and 16S8FE/B-GA1B [22] were used. For *Babesia* spp. and *Theileria* spp., the primers Nbab1F/Nbab1R [23] and RLBF2/RLBR2 [24] were used in the first and second PCR, respectively. The lamb samples that tested negative by microscopy and nested PCR were also analyzed by indirect enzyme-linked immunosorbent assay (iELISA). A recombinant protein of *B. ovis* Surface Antigen 1 (rBoSA1) was used as antigen in iELISA, as previously described [25]. However, due to the lack of commercially available and validated ELISA tests for detecting antibodies against *Theileria* spp. and *Anaplasma* spp. species in sheep, these pathogens were not tested using ELISA in this study. Five lambs and two goats were determined to be negative for *Babesia*, *Theileria*, and *Anaplasma* species, and were purchased and transported to Fırat University Animal Hospital for splenectomy (Figure 1a).

### 2.3. Splenectomy and Post-Operative Care

The lambs and goats underwent splenectomy using standard anesthesia, analgesic, and surgical techniques at the hospital [15,26]. After the surgery, they were transported to the small ruminant unit at the Ministry of Agriculture, Elazığ Veterinary Control Institute, Elazığ, Türkiye where the experiment would be conducted. Each animal was housed individually in a tick-free pen. For post-operative care, the animals received intravenous 0.9% physiological saline (250 mL) and 5% dextrose (250 mL) daily for two days to stabilize them. To prevent bacterial infections, a 5-day course of intramuscular oxytetracycline (Primamycin/LA^®^, Zoetis, Parsippany, NJ, USA) was administered. Additionally, the surgical wounds were treated daily with antibiotic spray (Neocaf, MSD, Rahway, NJ, USA, Oxytetracycline, aerosol spray suspension, 200 mL, USA) for a week. To prevent possible tick exposure, all experimental animals were treated with Flumethrin 1% pour-on (Ba-tick, BaVET, 10 mg Flumethrin, 100 mL, Istanbul, Türkiye) every 3 weeks throughout the study. The animals were fed twice a day with dry clover and crushed barley, with water available ad libitum. Two weeks after splenectomy, the goats and lambs were rechecked by microscopy and nested PCR, and it was confirmed that they were free of *Babesia*, *Theileria* and *Anaplasma* species.

### 2.4. Experimental Infections

In our recent study, we demonstrated that *B. aktasi* exhibits a high level of parasitemia and pathogenicity in immune-suppressed indigenous goats [15]. Therefore, immune-suppressed animals were used in the current study involving two separate experiments. In the first experiment, 2 splenectomized indigenous goats were used as donors (#Donor-1 and #Donor-2) to provide fresh blood infected with *B. aktasi* for infecting lambs. In the second experiment, 5 splenectomized Akkaraman breed lambs (#167, #281, #536, #585, #600) were used as recipient hosts (Figure 1b).

### 2.5. Inoculation of B. aktasi Stabilate in Donor Goats

#Donor-1 and #Donor-2 were each inoculated by intravenous injection of 15 mL *B. aktasi* stabilates, with 10% and 18% parasitemia, respectively. Following the stabilate inoculation, each donor received an intramuscular injection of dexamethasone (Vetakort^®^ 4 mg, Vetas, Türkiye; 20 mg daily dose) for 4 consecutive days [15,27]. From the first day post-inoculation (dpi) until the end of the clinical disease, the donors were monitored daily for clinical signs, and the following parameters were recorded: rectal temperature, presence of clinical symptoms of babesiosis (anemia, jaundice, and hemoglobinuria), parasitemia in Giemsa-stained smears prepared from the peripheral blood, and packed cell volume (PCV). When the parasitemia level reached 9.2% in #Donor-1 and 12% in #Donor-2, venous blood was collected from the donors, and immediately intravenously inoculated into the lambs (Figure 1b).

### 2.6. Inoculation of Fresh Blood Infected with B. aktasi in Recipient Lambs

The lambs #167 and #281 were each inoculated by intravenous injection with 15 mL of fresh blood infected with 9.2% parasitemia withdrawn from #Donor-1. Similarly, the lambs #536, #585, and #600 were inoculated with the same amount of fresh blood infected with 12% parasitemia withdrawn from #Donor-2 (Figure 1b). Following parasite inoculation, all lambs were administered intramuscular dexamethasone (Vetakort^®^ 4 mg, Vetas, Türkiye; 20 mg daily dose) for 4 consecutive days. They were monitored daily for evidence of clinical signs of babesiosis over 30 dpi. During this time, blood samples were collected daily from the jugular vein of lambs into vacuum tubes with and without EDTA for hemogram, DNA extraction, molecular detection and serum biochemistry. Peripheral thin blood smears were also prepared from the ear vein of the lambs for microscopic examination.

### 2.7. Microscopic Detection

Thin blood smears were fixed with methanol and stained with 10% Giemsa, then examined under a light microscope at 1000× magnification to detect intra-erythrocytic stages of *Babesia* spp. To measure parasitemia, blood smears from the animals’ ear tips were examined across at least 20 microscopic fields. Parasitemia, defined as the percentage of erythrocytes (PPE) infected with piroplasms, was calculated by dividing the number of parasitized erythrocytes by the total number of erythrocytes [15,26].

### 2.8. DNA Isolation, PCR and DNA Sequencing

Genomic DNA was extracted from whole blood samples using the PureLink™ Genomic DNA Mini Kit (Invitrogen Corporation, Carlsbad, CA, USA), following the manufacturer’s instructions. They were used as a template in the nested PCR for the amplification of *18S ribosomal RNA* gene of *Babesia*/*Theileria* species using primers Nbab-1F/Nbab-1R [23] and RLBF2/RLBR2 [24]. The PCR procedure and thermal cycling conditions were carried out as detailed by Ozubek et al. [15]. Positive (*B. aktasi* genomic DNA from GenBank accession no.MN559399.2) and negative (DNase/RNase-free water, and DNA obtained from a one-month-old lamb) controls were included in each PCR reaction. Ten microliters of the PCR products were electrophoresed on a 1.4% agarose gel for 30 min and visualized using the Quantum Vilber Lourmat (Marne-la-Vallee, France) gel imaging system.

To confirm the PCR results after parasite inoculation, the positive nested PCR products, which were detected in the recipient lambs, were subsequently purified using a PCR purification kit (Qiagen, Hilden, Germany). The amplicons were directly sequenced by the RLBF2/RLBR2 for the hypervariable V4 region of *18S rRNA* gene of *Babesia*/*Theileria* species [24]. The raw nucleotide sequences were visualized and evaluated using FinchTV 1.4.0. The sequences were analyzed for similarity by comparing them to those in the NCBI database using BLAST analysis.

## 3. Results

### 3.1. Babesia aktasi is Highly Virulent to Immune-Suppressed Indigenous Goats

**Figure 2:**
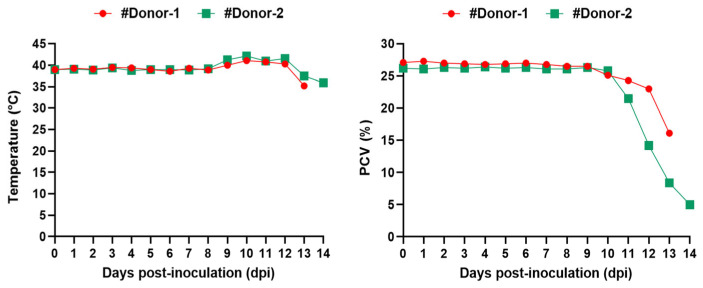
Body temperature and hematocrit (PCV) of donor goats experimentally inoculated by fresh blood infected with *Babesia aktasi*. #Donor-1 (●), #Donor-2 (■).

Following the stabilate inoculation, severe clinical babesiosis developed in both #Donor-1 and #Donor-2. The appearance of the first intra-erythrocytic parasites in thin blood smears was observed on the 4th and 5th dpi. Parallel to the increase in parasitemia, an increase in rectal temperature was observed, with the highest rectal temperatures recorded as 41.1 °C and 42.2 °C in #Donor-1 and #Donor-2, respectively. On the other hand, decreasing hematocrit (PCV) was observed in the donors (Figure 2 and Table 1). On the 12th dpi, approximately 9.2% and 12% parasitemia were detected in #Donor-1 and #Donor-2, respectively. On the same day, after blood was collected for the infecting lambs, imidocarb dipropionate (0.1 mg/kg body weight IM) and long-acting oxytetracycline (Primamycin/LA^®^, Zoetis, Parsippany, NJ, USA) (10 mg/kg body weight IM) were injected into the donors. However, the donors did not recover from the disease, and died on the 13th and 14th dpi. PCR followed by DNA sequencing confirmed that the clinical infection in the donor goats was caused by *B. aktasi*.

### 3.2. Babesia aktasi Failed to Infect Immune-Suppressed Sheep

**Figure 3:**
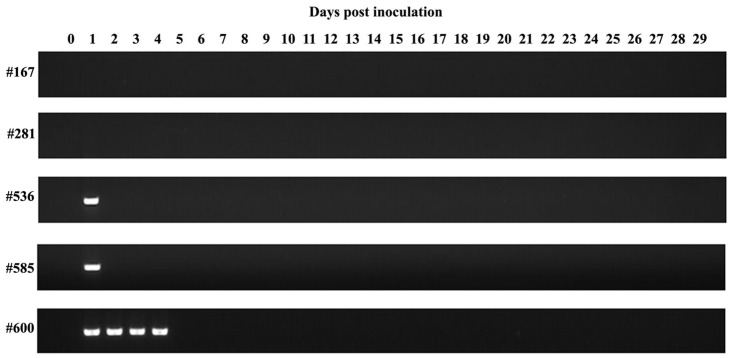
Gel image of the assay conducted to detect *Babesia/Theileria* species by targeting the *18S rRNA* gene using nested PCR in the recipient lambs (#167, #281, #536, #585, #600) inoculated with fresh blood infected with *Babesia aktasi*.

Following the inoculation of *B. aktasi*-infected blood, no clinical signs indicating the presence of babesiosis (fever, anemia, jaundice, and hemoglobinuria) were observed in the immune-suppressed recipient lambs over the monitoring period of 30 days. Rectal temperature and PCV were normal and stable. During this time, the intra-erythrocytic parasites of *B. aktasi* were not detected by microscopic examination of the Giemsa-stained blood smears. Surprisingly, no positive amplification products indicating the presence of the parasite DNA were also detected by nested PCR in the lambs #281 and #167 (Figure 3, Table 2). On the contrary, positive amplifications were detected in lambs #536 and #585 on the 1st dpi by nested PCR, and in lamb #600 up to the 4th day. However, these lambs tested negative by nested PCR in the subsequent days (Figure 3, Table 2).

To confirm the PCR results, partial nucleotide sequences of the *18S rRNA* gene from the positive PCR products detected in the recipient lambs were obtained and submitted to the GenBank database (accession no. PP980352-PP980354). BLAST analysis indicated that the sequences obtained in this study were 100% similar to the *Babesis* sp. Manay (*B. aktasi*) was isolated from a naturally infected indigenous goat (accession no. OM864353). Comparison of the sequences confirmed that the PCR positivity in the recipient lambs originated from the *B. aktasi* stabilate.

## 4. Discussion

Small ruminant babesiosis is a significant tick-borne protozoan disease caused by *Babesia* species, posing a major threat to sheep and goat health and productivity. Six *Babesia* species infective for cattle and buffalo are recognized as valid worldwide: *B. bigemina*, *B. bovis*, *B. divergens*, *B. major*, *B. ovata* and *B. orientalis* [28,29]. In contrast, *Babesia* species that infected small ruminants have not undergone such thorough taxonomic investigation. Until recently, three *Babesia* species infecting small ruminants (*B. ovis*, *B. motasi*, and *B. crassa*) have been recognized as valid species. However, in the past two decades, novel *Babesia* species/genotypes such as *Babesia* Xinjiang, *B. motasi*-like variants [*Babesia* sp. BQ1 (Lintan), *Babesia* sp. BQ1 (Ningxian)] and, finally, *B. aktasi*, have been reported [10,12,14,15,16,17,18,30]. Among these, *B. ovis* primarily affects sheep, causing clinical symptoms such as fever, anemia, jaundice, and hemoglobinuria, while *B. motasi* is more prevalent in goats, causing mild clinical manifestations [31]. Our previous field survey in the Mediterranean region of Türkiye revealed a high prevalence of *B. aktasi* in goats [19], while no molecular evidence of *B. aktasi* infection was found in sheep grazing in the same pasture [19]. However, this finding needed to be supported by in vivo experimental study. Therefore, we aimed to analyze the infectivity of *B. aktasi* in sheep through an experimental infection approach. Our experiments were performed on immune-suppressed lambs to increase the likelihood of infection, because the behavior of *B. aktasi* in sheep under field conditions remains unknown [15,27,32]. This novel parasite was isolated from a naturally infected asymptomatic local goat [14], and displayed typical clinical signs of caprine babesiosis in immune-suppressed indigenous goats [15]. In selecting donor and recipient lambs, we preferred those under one year of age that were negative for *Babesia*, *Theileria*, and *Anaplasma* species [15,27,32,33].

In a previous study determining the virulence of *Babesia* sp. Xinjiang in sheep, clinical and parasitological findings were observed in immune-suppressed lambs, while no findings were detected in spleen-intact lambs, except for the detection of parasite DNA using the molecular method (loop-mediated isothermal amplification) in the 2nd and 3rd weeks post parasite inoculation [27]. In the same study, an immune-suppressed calf was also infected with *Babesia* sp. Xinjiang, and monitored for 60 days; however, no piroplasm of the parasite were detected in the microscopic examination. These findings were suggested that *Babesia* sp. Xinjiang is infective for immune-suppressed sheep, but not infective for cattle [27]. In our study, immune-suppressed lambs were challenged with *B. aktasi* merozoites; however, no clinical and parasitological signs of infection were observed in any of the recipient lambs. Moreover, parasite DNA was not detected by nested PCR throughout the monitoring period of 30 days, except until the 4th day post parasite inoculation, due to the inoculum (Figure 3). These results indicated that *B. aktasi* is not infective for sheep, but PCR positivity may occur shortly after parasite inoculation, due to the inoculum.

Considering that sheep and goats are grazed together in the same pasture in the field, it is crucial to understand the impacts of sheep on the epidemiology of *B. aktasi*. The current study provides significant insights into the host specificity of the parasite. The results obtained in this study revealed that the recipient lambs inoculated by intravenous injection of fresh blood infected with *B. aktasi* displayed no clinical signs of infection. In contrast, the donor goats showed severe clinical signs and high parasitemia, indicating high virulence in immune-suppressed indigenous goats, similar to the results of the previous study with the same stabilate [15]. These findings are consistent with the previous studies documenting the fact that white-tailed deer (*Odocoileus virginianus*) and nilgai antelope (*Boselaphus tragocamelus*) did not show susceptibility to a strain of *B*. *bovis* that is highly pathogenic to cattle [33,34]. Our findings demonstrated the presence of potential species-specific immunity or resistance in sheep, and, importantly, it was observed independently of the immune system. This was specifically noted, as the study utilized splenectomized sheep, which lack a functional spleen and therefore have a compromised adaptive immune response. Host susceptibility to *Babesia* species is frequently observed. For instance, Chinese Tan sheep erythrocytes have been reported to be susceptible to *Babesia* sp. BQ1 (Lintan) infection, whereas French Vendéen sheep erythrocytes are resistant, highlighting breed-specific susceptibility [35]. Similarly, it has been reported that *B. bovis* does not cause clinical infection in spleen-intact water buffaloes, and they are able to significantly reduce circulating *B. bovis* parasites by an effective innate immune mechanism [36]. Together, these findings indicate that genetic and immunological factors inherent to each species or breed play an important role in influencing the outcome of *Babesia* infections. While the precise mechanisms behind species- or breed-specific resistance are not fully understood, this study suggests that they may involve non-adaptive immune responses or other genetic factors.

In this study, three out of five recipient lambs were found to be positive by nested PCR 1–4 dpi; however, these lambs tested negative by nested PCR in the following days. Contrary to our findings, three out of five water buffaloes inoculated with *B. bovis*-infected blood tested positive by nested PCR for 1–19 dpi [36]. The molecular findings obtained in this study suggest that there may be short-term positivity in some recipient hosts following the inoculation of *Babesia*-infected blood, due to the inocula. It is important to note that the host immune response can vary between pathogen delivery via needle inoculation and vector tick bite. Therefore, a limitation of our current study is the inability to perform a tick transmission experiment, as the tick vector of *B. aktasi* remains unknown. Another limitation of our study is that to eliminate the possibility of parasites at a level undetectable by nested PCR, the blood of nested PCR-negative recipient lambs could have been inoculated into immune-suppressed indigenous goats. However, it has been reported that when PCR-negative blood of nilgai antelope for *B. bovis* was inoculated into calves, no clinical and molecular findings were observed in the calves [33].

## 5. Conclusions

In conclusion, the findings of this experimental study revealed that sheep do not appear to be susceptible to infection by *B. aktasi*, which is highly pathogenic to immune-suppressed indigenous goats. This suggests that sheep do not play any role in the epidemiology of *B. aktasi*. This may be due to an innate immune response. Further research is needed to understand the underlying mechanisms of this species-specific susceptibility. Further tick transmission studies are needed to identify tick vectors involved in the transmission of *B. aktasi*. Additionally, considering the dense population of mountain goats that graze on the same pastures as domestic local goats, further research is necessary to identify the wild reservoir hosts of the parasite.

## Figures and Tables

**Table 1:** Clinical outcomes of donors following stabilate inoculation with *B. aktasi*.

Donor ID	Cryopreserved Stabliate	Prepatent Period	Max. Fever	Max. PPE	Clinical Signs	Response to Treatment	Death
#Donor-1	*B. aktasi* 10% PPE	4 dpi	41.1 °C	9.2%	+ *	-	+
#Donor-2	*B. aktasi* 12% PPE	5 dpi	42.2 °C	35%	+ *	-	+

* Severe anemia, high fever, jaundice, and hemoglobinuria. - The donors did not respond to the treatment. PPE: Percentage of parasitized erythrocytes.

**Table 2:** Clinical, microscopy, and nested PCR results in immune-suppressed lambs following the inoculation of fresh blood infected with *B. aktasi*.

Lamb ID	Source of Infection and Parasitemia (%)	Inoculum Amount and Route	Clinical Findings	Microscopic Examination	Nested PCR
#281	#Donor-1 (9.2%)	15 mL (iv)	-	-	-
#167	#Donor-1 (9.2%)	15 mL (iv)	-	-	-
#536	#Donor-2 (12%)	15 mL (iv)	-	-	+
#585	#Donor-2 (12%)	15 mL (iv)	-	-	+
#600	#Donor-2 (12%)	15 mL (iv)	-	-	+

## Data Availability

The raw data supporting the conclusions of this article will be made available by the authors on request.
